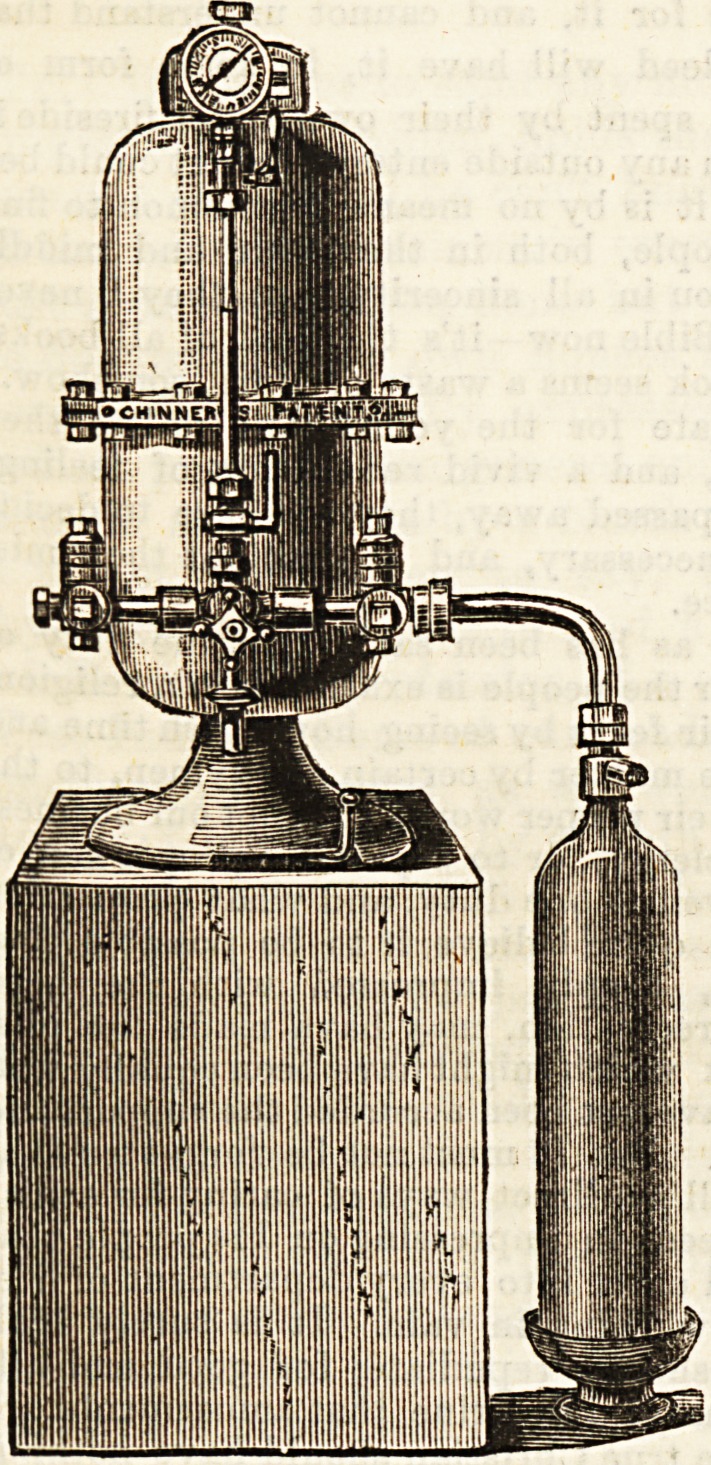# New Drugs, Appliances, and Things Medical

**Published:** 1891-01-24

**Authors:** 


### NEW DRUGS, APPLIANCES, AND THINGS MEDICAL.

[All preparations, appliances, novelties, etc., of which a notice is desired, should be sent for The Editor, to care of The Manager, 140, Strand, London, W.C.]

### AERATED WATERS FOR HOSPITALS.

It is not necessary to say to medical men and nurses that an abundant supply of all kinds of aërated waters of a good quality, and at a cheap rate, is a great desideratum in a hospital. Not only do many of the patients enjoy and benefit by aërated waters, but tired house doctors and nurses with impaired appetites both need and enjoy them, and should be able to obtain them without stint. But aërated waters, however obliging may be the manufacturer who supplies them, are a costly item, and have consequently to be indulged in sparingly when they ought to be made use of freely. The only way in which a sufficient supply can be provided for such a large institution as a hospital or a public school is that of preparing them on the premises. The raw materials from which aërated waters are made cost exceedingly little; it is the process of manufacture, which includes the preparation for the market, that is so expensive. What is wanted for the production of aërated water for hospitals, public institutions, and private families of the better class is an aërating machine, which shall be at once good, cheap, and easily worked. Such a machine has lately been put upon the market by Messrs. Chinnery and Co., of Leadenhall Buildings, London. "Chinnery's Patent," as the machine is called, seems to us exactly the kind of thing that is wanted. It is not only efficient, cheap, and easily worked, but quite portable. Perhaps the readiest way of showing its portability is to state that a specimen machine was carried by a workman to the office of The Hospital, and there the inventor, Mr. G. W. Chinnery, demonstrated its peculiar features one by one. The drawing, which we append, shows the general outlines of the machine, and gives an indication of its size and simplicity. The machine stands on a solid pedestal, and may be placed in the corner of any convenient room. The aërator is a vertical cylinder with a glass liner. It is made of gun metal, and tested to a high pressure. The carbonic acid gas is generated in the aërator when desired, and the cylinder can be attached to an ordinary water main by means of a union provided for that purpose, or the gas can be condensed in a separate cylinder, which, when attached to the machine, aërates the water or other fluids without the trouble of manipulating the acid and the alkali. A twelve pint machine, it is said, will make sixty dozen bottles of aërated water in a day; and one of the separate cylinders of thirty-three inches in height and five inches in diameter will, when fully charged, produce eight gross of carbonated water. This separate tube or cylinder can be charged at a cost of less than five shillings. It is easy, therefore, to calculate the price per bottle. The whole machine, with its additional charged tube, costs a very small sum, and can be readily used by inexperienced persons. It need not be attached to a water main, but can be easily employed in country houses, or for aërating distilled waters. Hospital officials will do well to avail themselves of Mr. G. W. Chinnery's readiness to explain his patent. If the machine proves as durable as it is simple and cheap, it will be an important addition to the daily resources of hospitals.

### PILLS.

Messrs. John Richardson and Co., Leicester, have forwarded to us samples of their coated pills. We find them to contain the ingredients that are ordered in the B.P. They are of excellent manufacture, pleasant to the eye and taste, and efficacious in use. That the coating does not interfere with their solubility is shown by the following test:—An artificial gastric fluid being made and kept at the temperature of 98·100 deg. Fahr., specimens of the pills were placed in it. with frequent agitation, the following appearances were noted—at the end of two minutes, the coating was swollen and cracking; at the end of three minutes, portions of the coating were detached; at the end of five minutes, the pill substance had begun to disintegrate. By the end of ten to twelve minutes the pill was a disorganized mass. The Blaud's pill was found to contain a dark coloured material which on the addition of water formed the well-known blue green precipitate formed by an alkaline carbonate with a ferrous salt. The pill was equally well made. On fracture the characteristic odours of peppermint and rhubarb were perceptible andthe taste was decidedly the combined nauseous bitter one of the various ingredients. On solution in water and the sediment being examined with a magnifying glass no coarse particles were seen. We have used this firm's pills for some years with great confidence, from the clinical results obtained. This confidence is strengthened by the recent investigation.

### "WARRINGTON" CHLOROFORM.

Mr. A. H. Mason, F.C.S., F.R.M.S. (Messrs. Seabury and Johnson, Jewin Street) has kindly supplied us with a specimen of the "Warrington" Chloroform. This product of modern chemistry is, we are informed, produced by the well-known "Ketone" process. We have compared the sample with another made by the ordinary process from pure alcohol by an eminent firm of chemists. It has answered all tests most satisfactorily. No odour is left on evaporating in the air from paper. No colouration with sulphuric acid. S.G. the same as guaranteed, neutral to test papers, and mixing in the proper proportions with the different substances we tried. We consider it a first-class sample of chloroform, and likely to stand well in the market, more especially as we believe it can be made almost as cheaply as the methylated varieties, and it certainly does not possess the disadvantages which some of these have.

### ROBIN'S READY-MADE LINSEED POULTICE.

This is a concentrated mucilaginous linseed preparation spread on an absorbent material, and having a gauze covering to go next to the skin. It is made in sheets, so that the exact size of the poultice can be cut off. It is always ready, and can have mustard or other medicants easily sprinkled on it. As it only takes three minutes soaking in hot water to prepare, is clean and does not smell sour (as some poultices from stale linseed meal do), and can be kept indefinitely till used, we think Messrs. Seabury and Johnson have introduced a decidedly valuable adjunct to the sick-room.

## Figures and Tables

**Figure f1:**